# Viscosity of Polycaprolactone Microplastic Dispersions and Nonlinear Kinetic Models of Plastic Fragmentation

**DOI:** 10.3390/molecules30102235

**Published:** 2025-05-21

**Authors:** Vincenzo Villani, Pier Luigi Gentili

**Affiliations:** 1 Department of Basic and Applied Sciences, University of Basilicata, Campus Macchia Romana, 85100 Potenza, Italy; 2 Department of Chemistry, Biology, and Biotechnology, University of Perugia, Via Elce di Sotto 8, 06123 Perugia, Italy; pierluigi.gentili@unipg.it

**Keywords:** plastic pollution, kinetic models of fragmentation, polycaprolactone (PCL), viscosity of dispersions, microplastics, nanoplastics, logistic equation, nonlinear kinetics

## Abstract

Viscosimetric experiments and microscopy measurements on microdispersions of polycaprolactone (PCL) plastics showed an unexpected exponential decrease in viscosity over the first 3 months and a plateau for a further 4 months of observations. This behavior is due to the release of nanoplastics from semicrystalline particles that reduce the viscosity of the dispersion, and leave stable and fine crystalline microplastics ranging in size from 30 to 180 μm. The development of nonlinear kinetic models for the fragmentation process from macro- to meso-, micro-, and nanoplastics reveals complex behavior that we call a cracking–leaching mechanism. The autocatalytic mechanical cracking of macroplastics larger than 5 mm is followed by a logistic-type mechanical cracking of mesoplastics between 5 and 1 mm. Therefore, microplastics smaller than 1 mm experience the leaching diffusion modeled via nonlinear coupled kinetic differential equations: semicrystalline microplastics quickly release nanoplastics from the amorphous fraction, followed by fine and stable crystalline microplastics. This proposed mechanism explains the size distribution of floating plastic debris in the oceans, with an unexpected gap of microplastics. Considering the outcome, a general reflection is made on the critical issues that currently appear unsolvable regarding plastic pollution.

## 1. Introduction

Global plastic production has reached an astonishing 413.8 Mt in 2023 [1]. Consequently, plastic pollution has become a global issue. Most plastic waste accumulates in landfills and natural environments. This raises concerns because it breaks down into small-sized plastic debris as a result of abrasion, fragmentation, and degradation [2,3]. Such plastic debris can easily spread uncontrollably across all environmental compartments, including water, sediment, soil, air, and biota. Fighting plastic pollution has become a complex challenge [4] because its environmental distribution must be monitored constantly. For hardly accessible niches, experimental and computational modeling has emerged as a viable monitoring approach.

The size distribution of plastic debris floating in the sea has been determined by extensive sampling of the surface waters of the oceans and the Mediterranean [5,6]. Research has highlighted the accumulation and the nearly uniform plastic pollution in the five large subtropical gyres, although the most serious accumulation of plastic debris is in the North Pacific Ocean [7].

Experiments on the fragmentation of polymer materials show that the size distribution of plastics (polymer fragments) conforms to a fractal process, spreading over several orders of magnitude [8]. Cracking patterns of photodegraded plastics are observed at multiple scales, from centimeters to a few microns [9]. Therefore, the fragmentation of the plastics into smaller and smaller pieces should lead to a rise in fragment count toward small sizes.

The density distribution of the oceans’ sampling data has a peak in the abundance of plastics around 2.2 mm and a pronounced gap below 1 mm. This is in contrast to the distribution expected from a fragmentation process without additional losses (conservative distribution). A Weibull distribution takes into account microplastic fragmentation [10].

The cumulative distribution of macroplastics N(l) (MacroPs, above 5 mm) aligns with a fractal process that has a power exponent λ=2.93, which is close to a three-dimensional fragmentation

$$N(l)=N_{0}\cdot l^{-\lambda }$$

$$\log(N)=\log(N_{0})-\lambda \cdot \log(l),$$

where l represents the linear dimension of MacroPs.

On the contrary, the deviation in mesoplastics (MesoPs, between 5 and 1 mm) from a power law and the disagreement for microplastics (MicroPs, smaller than 1 mm) are observed: fragments of a fine size become rare, although they should be prevalent if the plastics keep breaking down into smaller and smaller pieces (exponential fragmentation) [5,11,12,13].

Four possible interpretations have been proposed to explain the plastic losses (non-conservative distribution) in the MicroPs field: beach deposition, nano-fragmentation, biofouling, and ingestion by marine organisms [14,15,16,17,18].

We will show that the fragmentation of microplastics by means of a diffusion mechanism (which we call *leaching*) should be the primary process. The leaching of microplastics is due to the swelling and subsequent diffusion of NanoPs from the amorphous matrix as well as the final release of crystalline domains, which are smaller than the mesh size of 250 μm that is used in ocean water sampling.

Viscosimetric experiments were performed on polycaprolactone (PCL) microplastic dispersions. The PCL microplastics are semicrystalline polymers with a crystallinity of 42–56% depending on their thermal history and molecular weight [19].

The PCL fragmentation proceeds in several phases: (1) release of oligomers and NanoPs from amorphous regions, (2) crystalline MicroPs, and finally (3) NanoPs from crystalline regions. After about four months of abiotic hydrolytic degradation, a considerable amount of two small-sized plastic fractions were found and classified as submicron plastics (from 1 μm to 100 nm), nanoplastics (smaller than 100 nm), and oligomers [20].

In our study, viscosimetric experiments on PCL microplastic dispersions were performed, and nonlinear kinetic models of the fragmentation of MacroPs to MicroPs, NanoPs, and fine crystalline MicroPs are proposed.

## 2. Experimental Section I: Viscosity Measurements and Governing Equations

A dispersion of virgin PCL microplastics with particle dimensions smaller than 1 mm (CAPA 6800, PM = 7000, Solvay Belgium, Brussels, Belgium) was prepared in glycerol (1.2% by weight of particles per glycerol). The viscosimetric experiments were performed using a Couette viscometer (Anton Paar, Graz, Austria, ViscoQC 300) at 20 °C and at the slowest rotational rate of 0.01 rpm in order to approximate the zero-shear viscosity rate [21].

The dispersion of PCL microplastics in glycerol was kept thermostated at 20 °C for 7 months. Before each measurement, the dispersion was gently mixed. The measurements were taken during the 7-month period and the Newtonian viscosity was considered.

Measuring the viscosity of a dispersion of PCL microplastics in water is problematic as this system does not remain homogeneous for sufficiently long periods. Due to the difference in density (density of PCL with crystallinity = 0.50, ρ = 1.11 g/cm^3^) and the low viscosity of water (1.0 mPa·s at 20 °C), the heterogeneous phase rapidly separates from the liquid matrix. In contrast, glycerol offers the advantage of being a polar molecule that forms hydrogen bonds, with a high relative dielectric constant similar to that of water. However, with a viscosity 1000 times greater than that of water, it effectively slows down the separation of the dispersion and allows for meaningful viscosity measurements. In this way, it was possible to monitor the degradation of PCL microplastics dispersed in a polar, water-like liquid through viscometric analysis. The system can be a model of the polyester debris floating in water or, in general, of polymers with polar groups in an insoluble polar medium.

**Figure 1:**
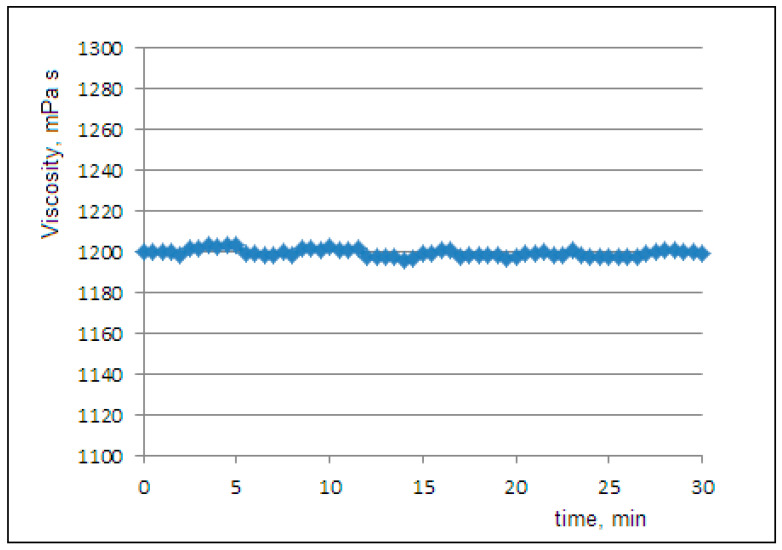
The Newtonian flow curve of pure glycerol at a rotational rate of 0.01 rpm and a temperature of 20 °C.

Figure 1 shows the viscosity curve at 20 °C of the pure glycerol that was used as the liquid phase in the PCL dispersion. We observed the expected Newtonian behavior and determined the value of $\eta_{0}=1200$ mPa·s.

**Figure 2:**
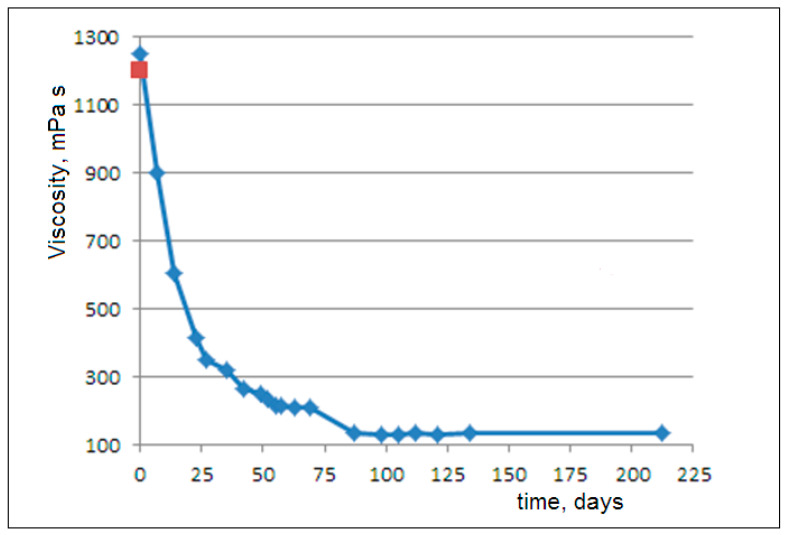
The viscosity trend of a PCL microplastic dispersion in glycerol over 212 days (7 months) (blue line) and the viscosity of pure glycerol (red point, from Figure 1) at a rotational rate of 0.01 rpm and a temperature of 20 °C.

In Figure 2, we observe an exponential decay of the viscosity after about 2 months of degradation. Then, a subsequent modest decrease occurs after about 1 month. Finally, an invariance in the viscosity is observed up to 7 months (3).

The observed exponential viscosity decay can be attributed to the release of nanoparticles from the amorphous matrix. As established in colloid science, nanodispersions can exhibit lower viscosity than both pure solvents and micro-scale dispersions at equivalent volume fractions. This reduction stems from the increased free volume between the nanoparticles and the dominant Brownian motion overpowering hydrodynamic interactions at the nanoscale [22,23,24,25,26,27,28,29,30].

The viscosity of a dilute microdispersion at time zero is described by the Einstein–Batchelor equation. At time zero, we observe the expected increase in viscosity $\eta_{\mu m}$ with respect to the value of the pure medium $\eta_{0}$ [22]

$$\eta_{\mu m}=\eta_{0}(1+k_{E}\cdot \varphi_{\mu m}),$$

where $\varphi_{\mu m}=\frac{\upsilon }{V}$ is the fraction of volume of dispersed microparticles υ in the volume of dispersion V and $k_{E}=\frac{1}{2}$.

The increased free volume $\upsilon_{free}$ between nanoparticles leads to a modified Einstein equation of the form

$$\eta_{nm}=\eta_{0}(1+k_{E}\frac{\upsilon -\upsilon_{free}}{V}).$$

When $\upsilon_{free}>\upsilon$, the viscosity of the nanodispersion becomes lower than that of the dispersing phase.

To compare the experimental viscosity curve with theoretical models, the weight concentrations in the dispersed phase must be converted into volume fractions in the continuous phase. Given the weight concentration C_0_ = 0.012 (dispersed phase/dispersing phase) of PCL (crystallinity 0.5, density $\rho_{PCL}=1.11$ g/cm^3^) in glycerol (density $\rho_{Gly}=1.26$ g/cm^3^), we can convert C_0_ to a volume fraction $\varphi_{0}=\frac{\upsilon_{PCL}}{V}$.

We then obtain the equation

$$\varphi_{0}=\frac{C_{0}\cdot \rho_{Gly}}{C_{0}\cdot \rho_{Gly}\;+\;\rho_{PCL}}=0.0134.$$

We can calculate the viscosity of the freshly prepared dispersion (at time zero) using Einstein’s equation, with the viscosity of glycerol $\eta_{0}=1200$ mPa·s, as

$$\eta (0)=\eta_{0}\cdot (1+2.5\cdot \phi_{0})=1239\;\mathrm{mPa}\cdot s.$$

This estimation matches well with the measured viscosity of 1248 mPa·s.

We account for the viscosity reduction by applying the modified Einstein equation

$$\eta_{nm}=\eta_{0}(1+2.5\frac{\upsilon -\upsilon_{free}}{V})=\eta_{0}\cdot (1+2.5\cdot \frac{\upsilon -\alpha \cdot \upsilon }{V}),$$

where the free volume scales with the dispersed phase volume as $\upsilon_{free}=\alpha \cdot \upsilon$. This leads to the equation

$$\eta_{nm}=\eta_{0}\cdot (1+2.5\cdot \varphi (1-\alpha )).$$

We analyzed the viscosity decay until convergence (reaching η=130 mPa·s after 100 days), starting from the zero-time value (η(0) = 1248 mPa·s). During this 100-day period, complete release of the amorphous phase was achieved. Given the initial PCL–glycerol concentration (0.012) with 0.5 crystallinity, as well as the densities of the amorphous phase ($\rho_{a}=1.10$ g/cm^3^), the crystalline phase ($\rho_{c}=1.22$ g/cm^3^), and the glycerol, the equilibrium released amorphous fraction is calculated as

$$\varphi_{a}\;=\;\frac{(1-X_{c})\cdot C}{\rho_{a}}÷(\frac{C}{\rho_{PCL}}+\frac{1}{\rho_{Gly}}),$$

where $\rho_{PCL}=X_{c}\rho_{c}+(1-X_{c}\rho_{a})$ is the average density of semicrystalline PCL. It follows that $\varphi_{a}=0.0068$.

Thus, for the steady-state viscosity, we write

$$\eta_{0}\cdot (1+2.5\cdot \varphi (1-\alpha ))=1248\cdot (1+2.5\cdot 0.0068\cdot (1-\alpha ))=130,$$

yielding α=53.69.

The determination of the parameter α enables us to calculate the volume fractions of the amorphous phase using the viscosity equation and the experimental viscosity values measured over time. We write the equation that directly relates the experimental viscosity to the amorphous volume fraction as

$$\eta =1248\cdot (1+2.5\cdot \varphi_{a}\cdot (1-53.69)).$$

**Figure 3:**
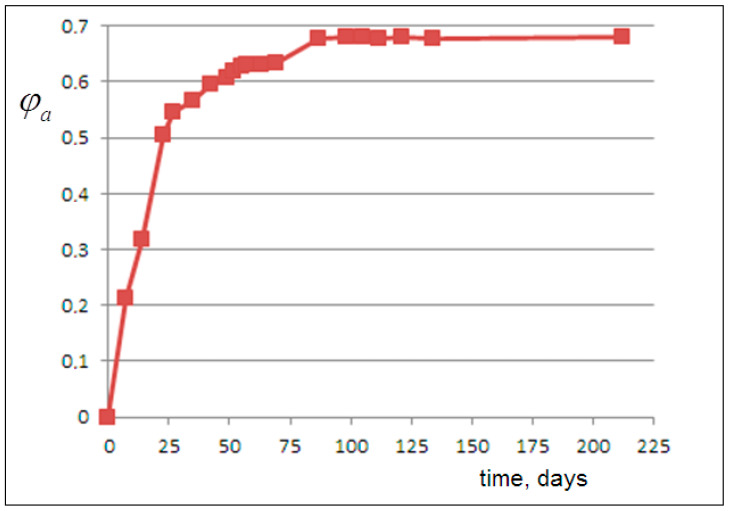
The volume fraction of the released amorphous phase, as derived from the data analysis of Figure 1 (see text for details).

From this, we derive, for each experimental viscosity value, the corresponding volume fraction of the released amorphous phase $\varphi_{a}$, plotted versus time in Figure 3. Then, the amorphous fraction curve $\varphi_{a}(t)$ can be matched with the NanoPs volume fraction predicted by the following kinetic models.

## 3. Experimental Section II: Microscopy Analysis

**Figure 4:**
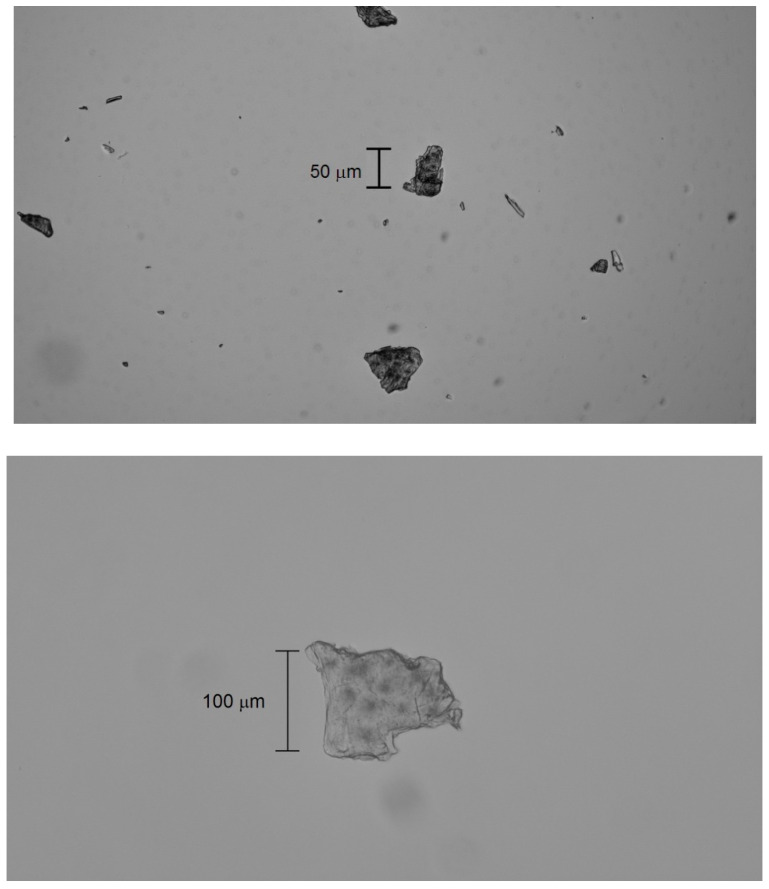
Bright-field microscopy with 4× (**top**) and 10× (**bottom**) objectives of fine MicroPs. The scale bar is shown in micrometers (µm).

Bright-field microscopy (Zeiss Axioskop 20 EL with digital camera, Oberkochen, Germany) revealed that the microplastics exhibit crystalline (with discernible circular crystallites), lamellar (flat and translucent with overlapping layers), and brittle (irregular edges and recognizable tiny flakes) morphologies (Figure 4).

**Figure 5:**
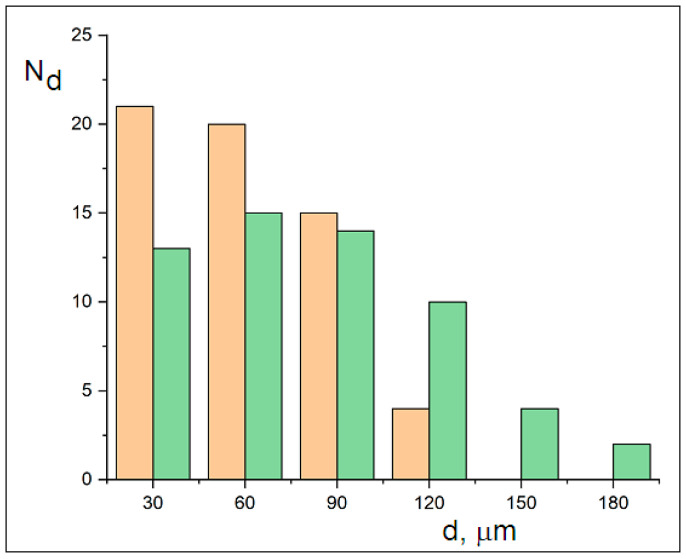
Histograms of the minimum (orange box, range: 30–120 µm) and maximum (green box, range: 30–180 µm) dimensions of crystalline microplastics after 7 months of dispersion in glycerol, measured via optical microscopy using a calibrated reticle.

Size distributions (measured using a system with a calibrated 10 µm reticle) of ~60 particles’ minimum and maximum dimensions were used to construct the histograms in Figure 5. The distributions follow Gaussian trends, with minimum dimensions ranging from 30 to 120 µm and maximum dimensions ranging from 30 to 180 µm. The width difference between the curves reflects mechanical cracking, while their Gaussian distribution indicates diffusive transport. The sizes of the crystalline MicroPs fall below the 250 µm sampling threshold for ocean water. This observed fragmentation into fine crystalline MicroPs (30–180 µm) may explain the reported microplastic sampling gap [5].

## 4. Modeling Section I: Kinetic Models of the Cracking Mechanism

The fracture mechanisms of marine plastics are not well known [8,31,32,33,34,35,36,37,38,39]. The proposed cracking–leaching mechanism of plastic debris fragmentation in water can be analyzed in three steps: from dispersions of MacroPs to MesoPs (*phase A*), from dispersions of MesoPs to semicrystalline MicroPs (*phase B*), and from dispersions of semicrystalline MicroPs to NanoPs and fine crystalline MicroPs (*phase C*). During *phase A*, the cracking mechanism of MacroPs>5 mm occurs, where mechanical cracks recursively generate increasingly smaller fragments from the initial $\alpha_{0}$ to $\beta_{0}$ over the time period $\tau_{0}$.

For *phase A*, we consider the power law distribution determined by Cózar et al. [5], with a spatial power law exponent λ=2.93 and the microscopic fragmentation of particles. In the standard case, the fragmentation velocity is proportional to the size of the produced particles. We have the following equation:

$$\frac{dl}{dt}=-k\cdot l,$$

where k is the microscopic fragmentation rate constant, representing the rate at which individual particles break apart. Thus, we obtain the exponential decay of particle size over time, where

$$l(t)=l_{0}\exp(-kt).$$

From the balance between the reduction in fragmentation rate and the increase in particle abundance, a first-order kinetic model emerges macroscopically for macroplastic fragmentation, along with an exponential growth in concentration α, where

$$\frac{d\alpha }{dt}=k_{1}\cdot \alpha .$$

The Smoluchowski fragmentation equation yields the macroscopic rate constant $k_{1}=\frac{k}{\lambda -1}$ by the ratio of the microscopic breakup constant k on λ−1=1.93. This evidence implies that some fragmentation events produce more than two fragments while others are binary, yielding a weighted average of 1.93 new particles per event.

The first-order autocatalytic kinetic differential equation, yielding the exponential analytical solution is the following:

$$\alpha (t)=\alpha_{0}\exp(k_{1}t).$$

At time $\tau_{0}$, we observe the MesoPs’ concentration $\beta_{0}$ as

$$\beta_{0}=\alpha_{0}\exp(k_{1}\tau_{0}).$$

Consistent with Cozar et al.’s [5] findings, the fragmentation kinetics change due to mechanical resistance in sufficiently small particles. Therefore, during *phase B* for MesoPs, we consider the microscopic fragmentation equation for particles of size l that includes the standard size-proportional term and an additional resistive term from cohesion energy, proportional to λ. This gives us

$$\frac{dl}{dt}=-k\cdot l+\frac{\lambda }{l}.$$

The term kl dominates in MacroPs, while $\frac{\lambda }{l}$ becomes significant for 1<l<5 mm in MesoPs. The cohesive forces originate from interactions between crystalline and amorphous domains within the semicrystalline particles. The equilibrium size is given by

$$\frac{dl}{dt}=-k\cdot l+\frac{\lambda }{l}=0,$$

from which we derive

$$l_{eq}=\sqrt{\frac{\lambda }{k}}.$$

Equilibrium occurs when cohesive forces balance fragmentation processes.

During *phase B*, we have a mechanical cracking-limited mechanism of mesoplastics, where 1<MesoPs<5 mm. The concentration of particles goes from $\beta_{0}\to B_{0}$. The increasing mechanical strength of smaller fragments acts as the dominant resistance factor against cracking, slowing the fragmentation. Then, the kinetics are described by a logistic differential equation whose analytical solution tends to a stationary value according to a sigmoid (for $\beta_{0}<B_{0}$) or an exponential decay (for $\beta_{0}>B_{0}$), where the particles are reducing to microplastics with an average size of $l_{eq}=1$ mm.

$$\frac{d\beta }{dt}=k_{2}\beta -k_{-2}\beta^{2}$$

$$\beta (t)=\frac{\beta_{\infty }}{1+\frac{\beta_{\infty }-\beta_{0}}{\beta_{0}}\cdot \exp(-k_{2}\cdot t)}$$

$$\beta_{\infty }=\frac{k_{2}}{k_{-2}}\beta_{0}=B_{0}$$

When dispersed particles approach MicroPs around 1 mm or less, their enhanced mechanical strength suppresses cracking, causing diffusion of the dominant mass transport mechanism.

Cracking (*phase A* and *B*) is one process in a series of processes since there is a high prevalence of mechanical fragmentation over chemical diffusion (high volume–surface ratio). The subsequent dispersion of MicroPs during leaching is a diffusive transport process in which multiple variables are coupled. In fact, during *phase C*, the difference in behavior of the amorphous and crystalline polymer fractions becomes relevant: the amorphous fraction with a lower density than the crystalline fraction experiences swelling of the polar medium, which facilitates the release of NanoPs. The dissolution of the binding amorphous matrix involves the release of semicrystalline sub-MicroPs that accelerate the dissolution of the amorphous matrix, and so on. This is the two-variable leaching model of MicroPs. Finally, even the sub-MicroPs of higher crystallinity undergo a slower dissolution into crystalline MicroPs. Fine crystalline MicroPs are stable and remain in the medium for long periods of time. This is the MicroPs leaching model with three variables that are coupled. In the next paragraph, the leaching models of increasing complexity with two or three variables are examined.

## 5. Modeling Section II: Kinetic Models of the Leaching Mechanism

**Figure 6:**
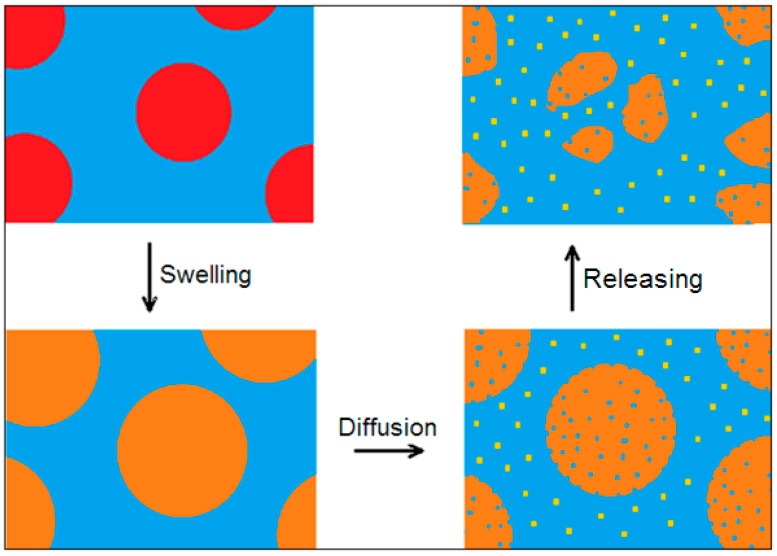
The leaching mechanism of MicroPs’ fragmentation, which involves the swelling of the amorphous matrix, the diffusion of NanoPs from the amorphous matrix, and the releasing of MicroPs into sub-MicroPs of higher crystallinity.

The leaching mechanism of MicroPs’ fragmentation is analyzed using two- or three-variable models. From the kinetic point of view, at first, the transport of matter occurs through nanoplastic diffusion from the amorphous phase that has undergone swelling. As the NanoPs are released from the amorphous phase, the progressively increasing porosity of microplastics enhances diffusive transport, creating a self-reinforcing mechanism. Since the amorphous matrix serves as the binder between crystalline domains, sub-MicroPs are released into the fluid medium (Figure 6).

We represent the leaching mechanism as the swelling, diffusion, and release processes of microplastics by means of two nonlinear, coupled, logistic-like equations. In this model, the diffusion of NanoPs C is logistic with a feedback action due to the coupling with sub-MicroPs B, made up of semicrystalline domains bound by the amorphous matrix. Furthermore, the cracking of MicroPs B is logistic with a negative feedback action due to the coupling with C. The forward rate constants satisfy the relationship k_1_ ≫ k_2_ to ensure dominant production of NanoPs. Therefore, we obtain the following equation:

$$\frac{dB}{dt}=k_{1}B-k_{-1}BC$$

$$\frac{dC}{dt}=k_{2}C-k_{-2}CB.$$

Concentrations are expressed as volume fractions of the dispersed phase. To perform fitting between calculated C(t) and experimental $\varphi_{a}(t)$ values, we scale the time t of the viscosity experiments (based on 212 days of observations) to the interval [0,1], where

$$\tau =\frac{t-t_{\min}}{t_{\max}-t_{\begin{array}{l} \min \end{array}}}=\frac{t}{212}.$$

Given the initial condition B(0)=1,C(0)≈0 and the boundary condition of total volume fraction conservation B(t)+C(t)=1, we set k_1_ = 0 (no autoproduction of B), k_2_ > k_−2_ (B production dominates over inhibition), and k_−1_ > 0 (B decay occurs).

**Figure 7:**
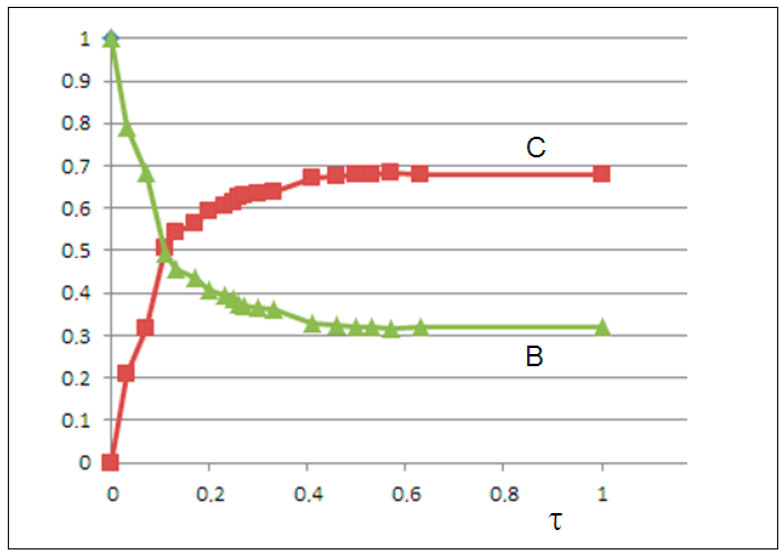
The solutions of the two-variable model: the time decay of semicrystalline microplastics B(t) (green line) and the growth of nanoplastics C(t) (red line) vs. the scaled time τ.

We optimized the kinetic parameters by minimizing the mean squared error (MSE) between calculated C(t) and experimental $\varphi_{a}(t)$ values. Solving the differential equations yielded an excellent fit with MSE = 0.0001, whereby we obtained the following rate constants: k_1_ = 0, k_−1_ = 1.9, k_2_ = 2.3, and k_−2_ = 1.2. The growth of C(t) and the decay of B(t) are shown in Figure 7.

To model the disaggregation of semicrystalline sub-microparticles (sub-MicroPs) B into crystalline microparticles (crystalline MicroPs) D, we developed a system of coupled nonlinear differential equations with three variables, where

$$\frac{dB}{dt}=k_{1}B-k_{-1}BC-k_{-1}BD$$

$$\frac{dC}{dt}=k_{2}C-k_{-2}CB+k_{-2}CD$$

$$\frac{dD}{dt}=k_{3}D-k_{-3}DB.$$

To account for volume fraction conservation B(t)+C(t)+D(t)=1 in the kinetic constant calculations, we reformulate the equations as follows:

$$\frac{dB}{dt}=-k_{-1}BC-k_{-2}B(1-B-C)$$

$$\frac{dC}{dt}=k_{2}C-k_{-2}BC$$

D(t)=1−B(t)−C(t).

Therefore, the kinetics of D(t) follow the form

$$\frac{dD}{dt}=-\frac{dB}{dt}-\frac{dC}{dt,}$$

yielding

$$\frac{dD}{dt}=k_{1}BC+k_{-2}BD-k_{2}C+k_{-2}CB.$$

To be consistent with the original equation for D, we derive

$$k_{3}D+k_{-3}BD=k_{1}BC+k_{-2}BD-k_{2}C+k_{-2}CB.$$

This yields the system of kinetic equations for B, C, and D that satisfies the total volume fraction conservation:

$$\frac{dB}{dt}=-k_{-1}BC-k_{-2}B(1-B-C)$$

$$\frac{dC}{dt}=k_{2}C-k_{-2}BC$$

$$k_{3}D+k_{-3}BD=k_{1}BC+k_{-2}BD-k_{2}C+k_{-2}CB.$$

Starting from the initial condition B(0)=1,C(0)≈0,D(0)≈0, in order to ensure B(t) decreases monotonically while C(t) and D(t) increase, we set k_1_ = 0 (no autoproduction of B), k_2_ > k_−2_ (B production dominates over inhibition), k_−1_ > 0 (B decay occurs), and k_3_ > k_−3_ (D production dominates over inhibition).

**Figure 8:**
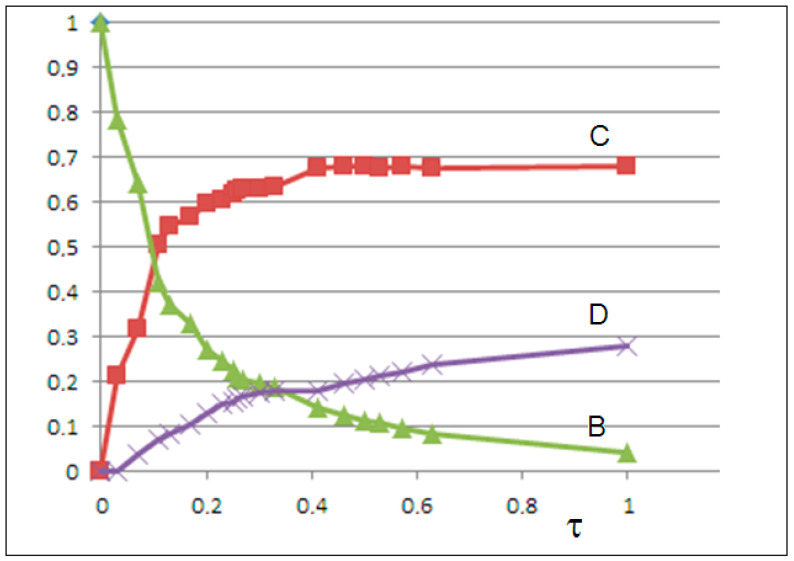
The solutions of the three-variable model: the time abundance of semicrystalline microplastics B(t) (green line), nanoplastics C(t) (red line), and crystalline microplastics D(t) (purple line) vs. the scaled time.

By solving the differential equations and minimizing the mean squared error (MSE) between calculated C(t) and experimental $\varphi_{a}(t)$ values, we obtain the rate constants k_1_ = 0, k_−1_ = 0.62, k_2_ = 1.35, k_−2_ = 0.25, k_3_ = 0.42, and k_−3_ = 0.08, with MSE = 0.0012, indicating excellent agreement. The solutions for B(t), C(t), and D(t) are shown in Figure 8.

In both models, in addition to the logistic growth of the NanoPs C, we observe the logistic decay of the semicrystalline MicroPs B. In the second model, the growth of the fine crystalline microPs D occurs at the expense of the decay of B particles.

In summary, the cracking model explaining the fragmentation of MacroPs and MesoPs determined above takes into account the sampling data from Cózar [5], and adds in the leaching–cracking model with the logistic growth of NanoPs, the subsequent decay of the semicrystalline MicroPs, and the logistic growth of the crystalline MicroPs, consistent with the study by Fernández-Piñas [20], the experimental viscosimetric data and the microscopy analysis.

## 6. Conclusions

Viscosimetric experiments, microscopy analysis, and modeling demonstrate that the degradation of PCL polyester microplastics in a polar dispersing medium proceeds rapidly enough to release the amorphous phase as nanoplastics. These nanoplastics are unstable semicrystalline microplastics and fine crystalline microplastics that persist long-term in the medium.

The experimental data were interpreted by means of nonlinear kinetic models of increasing complexity. The dispersion of macroplastics was interpreted by means of an autocatalytic mechanism of mechanical cracking with exponential growth. The dispersion of mesoplastics occurred by means of a logistic mechanism of mechanical cracking, with negative feedback due to the progressively smaller size of the released fragments.

The dispersion of microplastics was modeled by means of two- or three-variable diffusive kinetic mechanisms. The semicrystalline composition of the microplastics was taken into account. Next, the leaching mechanism with two coupled variables considered the particles’ release from the amorphous fraction and the crystalline fraction. The amorphous matrix underwent swelling and quickly released particles in the form of nanoparticles in the first stage. The increase in nanoparticle concentration caused the observed decrease in viscosity, a result that is in agreement with experimental data and theoretical frameworks. Subsequently, semicrystalline sub-microplastics, held together by the amorphous matrix, were released and the concentration of microplastics decayed until it reached a plateau. Finally, we considered the leaching model with three coupled equations in which the kinetics of three interacting species were present: nanoplastics, semicrystalline microplastics, and crystalline microplastics. The nanoplastics were released exponentially from the amorphous matrix and then the microplastics disaggregated into semicrystalline sub-microplastics. Finally, fine and stable crystalline microplastics were released at the expense of the semicrystalline particles. The final crystalline particles only very slowly disaggregated further. In this way, the abundance of the unstable semicrystalline MicroPs decreased at the expense of the stable crystalline MicroPs, explaining the invariance of the viscosity observed over long times.

Microscopic observations showed that the final crystalline MicroPs had a size between 30 and 180 μm, smaller than the sampling threshold (250 μm) used in oceanographic studies. Therefore, the observed reduction in abundance of MicroPs in water samples would be due to the degradation of semicrystalline MicroPs into NanoPs and fine and stable crystalline MicroPs that escape sampling.

Therefore, the kinetic equations of the cracking process of MacroPs α(t) and MesoPs β(t), and the leaching process of semicrystalline MicroPs B(t), NanoPs C(t), and crystalline MicroPs D(t), with the initial condition of $B_{0}=1$ and the boundary condition of B(t)+C(t)+D(t)=1, are as follows:

$$\frac{d\alpha }{dt}=k_{1}\alpha$$

$$\frac{d\beta }{dt}=k_{1}\beta -k_{-1}\beta^{2}$$

$$\frac{dB}{dt}=-k_{-1}BC-k_{-1}BD$$

$$\frac{dC}{dt}=k_{2}C-k_{-2}CB+k_{-2}CD$$

$$\frac{dD}{dt}=k_{3}D-k_{-3}DB.$$

The outcome warns against the insidious behavior of semicrystalline polymers dispersed in water that degrade into NanoPs as well as stable and fine crystalline MicroPs that have a lifespan long enough to enter the food chain and reach humans. This process is widely documented.

NanoPs and crystalline MicroPs escape water sampling and would be the most insidious particles produced by the fragmentation of dispersed plastics, even of the biodegradable type [20]. These are critical issues that cannot be resolved a posteriori; they require preventive interventions.

The kinetic fragmentation mechanism has very general characteristics. Although it was developed for a polyester, it should nonetheless be representative of all semicrystalline polymers with polar backbones (e.g., polyamides). Additionally, multiple more complex behaviors arise, such as the behavior of dispersions of nonpolar polymers (e.g., polyolefins); the role of microbial activity developing in seawater; the effects of physical (thermal–UV) and chemical (oxidation–hydrolysis) degradation; the role of biofilm and biofouling formation; and the degradation of fine crystalline microplastics. These increasing levels of complexity can be incorporated into the kinetic model by accounting for additional equations, considering feedback, and optimizing the kinetic constants.

Plastic pollution is a plague of modern society as stated in the Introduction and the related bibliography. The project that aims to replace the linear economy of polymeric materials with a circular economy has not yielded the desired results: plastic waste collected for recycling worldwide is only 15% [40]. In any case, recycling can be repeated a limited number of times before giving rise to a low molecular weight material that is no longer usable and that eventually ends up in a landfill or in the environment. Furthermore, it is difficult to have a single-component recycled material that is desirable. An improvement in this sense could include using single-component materials or combinations of compatible polymer materials in the production of plastic objects that give a stable and useful polymer blend.

In conclusion, the use of biodegradable polymers or the recycling of plastic material is not enough. Indeed, this process can be an additional source of microplastic production. When collected, plastics must be sorted by type, washed, and shredded. These processes waste resources and contaminate water. They also create microplastics and nanoplastics [41]. It should be noted that the remediation of ocean gyres (and waters in general) is not practised because it is not economically sustainable. Regardless, NanoPs and fine crystalline MicroPs would still remain elusive. We recall that the sustainability criterion, based on the three pillars of environmental, social, and economic compatibility, does not prioritize the needs of the environment and society over economic needs [42,43]. An ethical choice (for the benefit of the common good) can be deemed “not economically sustainable” and therefore, it will not be implemented while a completely sustainable solution is waited for.

Faced with the unsolvable critical issues of elusive plastics, which are not sustainable from an environmental and social point of view, a preventive ethical choice that prioritizes the environment and society is necessary, such as the ban on nonessential, single-use plastics.

## Data Availability

Data are contained within the article.

## References

[1] Published by Statista Research Department. https://www.statista.com/statistics/282732/global-production-of-plastics-since-1950/.

[2] Thompson R.C., Olsen Y., Mitchell R.P., Davis A., Rowland S.J., John A.W.G., McGonigle D., Russell A.E. (2004). Lost at sea: Where is all the plastic?. Science.

[3] Bao L.-J., Mai L., Liu L.-Y., Sun X.-F., Zeng E.Y. (2024). Microplastics on the Planet: Current Knowledge and Challenges. Environ. Sci. Technol. Lett..

[4] Gentili P.L. (2021). Why is Complexity Science valuable for reaching the goals of the UN 2030 Agenda?. Rend. Lincei Sci. Fis. E Nat..

[5] Cózar A., Echevarría F., González-Gordillo J.I., Irigoien X., Ubeda B., Hernández-León S., Palma Á.T., Navarro S., García-De-Lomas J., Ruiz A. (2014). Plastic debris in the open ocean. Proc. Natl. Acad. Sci. USA.

[6] Cózar A., Sanz-Martín M., Martí E., González-Gordillo J.I., Ubeda B., Gálvez J.Á., Irigoien X., Duarte C.M. (2015). Plastic Accumulation in the Mediterranean Sea. PLoS ONE.

[7] Andrady A.L. (2011). Microplastics in the marine environment. Mar. Pollut. Bull..

[8] Aoki K., Furue R. (2021). A model for the size distribution of marine microplastics: A statistical mechanics approach. PLoS ONE.

[9] Barnes D.K.A., Galgani F., Thompson R.C., Barlaz M. (2009). Accumulation and fragmentation of plastic debris in global environments. Phil. Trans. R. Soc. B.

[10] Wang L., Li P., Zhang Q., Wu W.-M., Luo J., Hou D. (2021). Modeling the Conditional Fragmentation-Induced Microplastic Distribution. Environ. Sci. Technol..

[11] Isobe A., Kubo K., Tamura Y., Nakashima E., Fujii N. (2014). Selective transport of microplastics and mesoplastics by drifting in coastal waters. Mar. Pollut. Bull..

[12] Isobe A., Uchida K., Tokai T., Iwasaki S. (2015). East Asian seas: A hot spot of pelagic microplastics. Mar. Pollut. Bull..

[13] Eo S., Hong S.H., Song Y.K., Lee J., Shim W.J. (2018). Abundance, composition, and distribution of microplastics larger than 20 μm in sand beaches of South Korea. Environ. Pollut..

[14] Reisser J.W., Slat B., Noble K.D., Plessis K.D., Epp M., Proietti M.C., de Sonneville J., Becker T., Pattiaratchi C. (2015). The vertical distribution of buoyant plastics at sea: An observationalstudy in the North Atlantic Gyre. Biogeosciences.

[15] Enders K., Lenz R., Colin Stedmon A., Torkel Nielsen G. (2015). Abundance, size and polymer composition of marine microplastics 10 μm in the Atlantic Ocean and their modelled vertical distribution. Mar. Pollut. Bull..

[16] Kooi M., Koelmans A.A. (2019). Simplifying microplastic via continuous probability distributions for size, shape, and density. Environ. Sci. Technol. Lett..

[17] Lindeque P.K., Cole M., Coppock R.L., Lewis C.N., Miller R.Z., Watts A.J., Wilson-McNeal A., Wright S.L., Galloway T.S. (2020). Are we underestimating microplastic abundance in the marine environment? A comparison of microplastic capture with nets of different mesh-size. Environ. Pollut..

[18] Tokai T., Uchida K., Kuroda M., Isobe A. (2021). Mesh selectivity of neuston nets for microplastics. Mar. Pollut. Bull..

[19] Tenorio-Alfonso A., Vázquez Ramos E., Martínez I., Ambrosi M., Raudino M. (2023). Assessment of the structures contribution and mechanical properties of polycaprolactone/pluronic blends. J. Mech. Behav. Biomed. Mater..

[20] Tamayo-Belda M., Pulido-Reyes G., González-Pleiter M., Martín-Betancor K., Leganés F., Rosal R., Fernández-Piñas F. (2022). Identification and toxicity towards aquatic primary producers of the smallest fractions released from hydrolytic degradation of polycaprolactone microplastics. Chemosphere.

[21] Villani V. (2024). Viscosity Flow Curves of Agar and the *Bounded Ripening Growth* Model of the Gelation Onset. Molecules.

[22] Batchelor G.K. (1977). The effect of Brownian motion on the bulk stress in a suspension of spherical particles. J. Fluid Mech..

[23] Villani V., Lavallata V. (2019). Rheology of lightly-cured polydimethylsiloxane liquid blends. Eur. Polym. J..

[24] Villani V., Lavallata V. (2018). Unexpected rheology of polydimethylsiloxane liquid blends. Macromol. Chem. Phys..

[25] Villani V., Lavallata V. (2017). Unusual rheological properties of lightly crosslinked polydimethylsloxane. Macromol. Chem. Phys..

[26] Mackay M.E., Dao T.T., Tuteja A., Ho D.L., Van Horn B., Kim H.-C., Hawker C.J. (2003). Nanoscale effects leading to non-Einstein-like decrease in viscosity. Nat. Mater..

[27] Jain S., Goossens J.G.P., Peters G.W.M., van Duin M., Lemstra P.J. (2008). Strong decrease in viscosity of nanoparticle-filled polymer melts through selective adsorption. Soft Matter.

[28] Huang H., Li B., Simien C.E., Simien D.O. (2017). Viscosity and morphology modification of length sorted single-walled carbon nanotubes in PIB matrices. J. Nanomater..

[29] Tuteja A., Duxbury P.M., Mackay M.E. (2007). Multifunctional Nanocomposites with Reduced Viscosity. Macromolecules.

[30] Tan H., Xu D., Wan D., Wang Y., Wang L., Zheng J., Liu F., Ma L., Tang T. (2013). Melt viscosity behavior of C60 containing star polystyrene composites. Soft Matter.

[31] Tanaka H., Inaba S., Nakazawa K. (1996). Steady-state size distribution for the self-similar collision cascade. Icarus.

[32] Kolmogorov A. (1941). On the logarithmic normal distribution of particle sizes under grinding. Dokl. Akad. Nauk SSSR.

[33] Middleton G. (1970). Generation of the log-normal frequency distribution in sediments. Topics in Mathematical Geology.

[34] Crow E.L., Shimizu K. (1988). Lognormal Distributions Theory and Applications.

[35] Vincent P. (1986). Differentiation of modern beach and coastal dune sands—A logistic regression approach using the parameters of the hyperbolic function. Sediment. Geol..

[36] Kobayashi N., Kohyama K., Sasaki Y., Matsushita M. (2006). Statistical laws for food fragmentation by human mastication. J. Phys. Soc. Jpn..

[37] Ishii T., Matsushita M. (1992). Fragmentation of Long Thin Glass Rods. J. Phys. Soc. Jpn..

[38] Weibull W. (1951). A Statistical Distribution Function of Wide Applicability. J. Appl. Mech..

[39] Brown W.K., Wohletz K.H. (1995). Derivation of the Weibull distribution based on physical principles and its connection to the Rosin–Rammler and lognormal distributions. J. Appl. Phys..

[40] Published by Statista Research Department. https://www.statista.com/topics/5401/global-plastic-waste/.

[41] Published by PlasticPollutionCoalition. https://www.plasticpollutioncoalition.org/blog/2024/6/4/plastic-recycling-is-a-false-solution-to-plastic-pollution.

[42] Cheever F., Dernbach J.C. (2015). Sustainable Development and Its Discontents, University of Denver Digital Commons @ DU Sturm College of Law: Faculty Scholarship. https://digitalcommons.du.edu/law_facpub.

[43] Gentili P.L., Cardinali G., Dominici P., Grohmann D., Menconi M.E., Santi C. The science of complex systems for preparing the new generation to tackle global challenges. Proceedings of the 8th International Conference on Higher Education Advances (HEAd’22).

